# Text Book on Surgery

**Published:** 1887-06

**Authors:** 


**Text Book on Surgery**— General, Operative and Mechanical. By John A. Wyeth, M. D., Professor of Surgery in the New York Polyclinic, Surgeon to Mt. Sinai Hospital. New York: D. Appleton & Co. 1887.

A book without either preface or introduction, but every page of its 777 is worth reading. A surgeon who has in his library such works as Agnew's, Gross', Hamilton's, Ashurst's System, etc, may think that he needs no new one, but we venture the opinion that if he sees this work he will buy it. The first thing that will attract him is the elegant colored illustrations — the new cuts and the perfect press-work. Then the terseness and vigor of the style in which it is written will delight him, and a more complete examination will convince him that the book is so full of meat that he cannot do without it. It is, too, a book excellently well adapted to the use of the student.

